# Transfer of MicroRNA-216a-5p From Exosomes Secreted by Human Urine-Derived Stem Cells Reduces Renal Ischemia/Reperfusion Injury

**DOI:** 10.3389/fcell.2020.610587

**Published:** 2020-12-22

**Authors:** Yinmei Zhang, Junxiong Wang, Boxin Yang, Rui Qiao, Aiwei Li, Han Guo, Jie Ding, Hui Li, Hong Ye, Di Wu, Liyan Cui, Shuo Yang

**Affiliations:** ^1^Department of Laboratory Medicine, Peking University Third Hospital, Beijing, China; ^2^Department of Gastroenterology, Beijing Friendship Hospital, Capital Medical University, Beijing, China

**Keywords:** acute kidney injury, human urine-derived stem cells, exosomes, miR-216a-5p, phosphatase and tensin homolog

## Abstract

Human urine-derived stem cells (USCs) protect rats against kidney ischemia/reperfusion (I/R) injury. Here we investigated the role of USCs exosomes (USCs-Exos) in protecting tubular endothelial cells and miRNA transfer in the kidney. Human USCs and USCs-Exos were isolated and verified by morphology and specific biomarkers. USC-Exos played a protective role in human proximal tubular epithelial cells (HK-2) exposed to hypoxia/reoxygenation (H/R). USCs-Exos were rich in miR-216a-5p, which targeted phosphatase and tensin homolog (PTEN) and regulated cell apoptosis through the Akt pathway. In HK-2 cells exposed to H/R, incubation with USC-Exos increased miR-216-5p, decreased PTEN levels, and stimulated Akt phosphorylation. Exposure of hypoxic HK-2 cells to USCs-Exos pretreated with anti-miR-216a-5p can prevent the increase of miR-216-5p and Akt phosphorylation levels, restore PTEN expression, and promote apoptosis. The dual-luciferase reported gene assay in HK-2 cells confirmed that miR-216a-5p targeted PTEN. In rats with I/R injury, intravenous infusion of USCs-Exos can effectively induce apoptosis suppression and functional protection, which is associated with decreased PTEN. Infusion of exosomes from anti-miR-216a-5p-transfected USCs weakened the protective effect in the I/R model. Therefore, USCs-Exos can reduce renal I/R injury by transferring miR-216a-5p targeting PTEN. Potentially, USCs-Exos rich in miR-216a-5p can serve as a promising therapeutic option for AKI.

## Introduction

Renal ischemia/reperfusion (I/R) injury is one of the most common causes of acute kidney injury (AKI) and is strongly associated with tubular cell necrosis and endothelial cell dysfunction (Cui et al., 2011; Sharfuddin and Molitoris, 2011; Borisov and Shilov, 2017). However, the underlying mechanism of sudden loss of renal function and tissue damage has not been fully elucidated, leading to limited treatment and high mortality.

It has been reported that some somatic stem cells applied in various studies could enhance functional recovery and pathological morphology improvement in patients with AKI (Tetta et al., 2020), but some of these stem cells are obtained through invasive procedures which may be harmful to patients. Therefore, we need to find a more feasible, non-invasive, and effective treatment strategy for AKI. Urine-derived stem cells (USCs) are emerging as a promising cell resource for their multiple applications in cell therapy and tissue engineering due to their non-invasive obtaining procedure, potent proliferation capabilities, and ability to serve as original cells for reprogramming into disease-specific iPSCs (Ji et al., 2017; Wu et al., 2019). In addition, since USCs are highly homologous with the urinary system, they have a promising potential in bladder tissue regeneration and urethral reconstruction and can effectively reduce inflammation and fibrosis (Yang et al., 2018). Shou Fu et al. suggested that USCs may promote kidney repair and improve function following ischemic AKI (Tian et al., 2017).

The paracrine mechanism of exosomes is one of the main mechanisms of stem cell therapy for AKI. Exosomes are vesicles with a bilayer membrane structure, about 30–120 nm in diameter, which are released into extracellular spaces or biological body fluids by exocytosis. Exosomes are rich in proteins, mRNAs, miRNAs, and other molecules. As carriers, they are taken up by renal tubular epithelial cells, macrophages, and endothelial cells and can participate in the regulation of the pathological process of renal I/R injury, such as cell proliferation, autophagy, apoptosis, oxidative stress, inflammatory response, angiogenesis, fibrosis, and immune escape. *In vivo* and *in vitro* studies have shown that exosomes derived from different types of stem cells could effectively protect against AKI (Grange et al., 2019). Exosomes can affect receptor cells through a variety of mechanisms, such as interacting with surface receptors and transporting proteins, nucleic acids (mRNA and miRNA), and other active substances.

miRNAs carried in exosomes may play a key role in the regulation of kidney I/R injury and the treatment of AKI. De Almeida et al. identified that mesenchymal stromal cells (MSCs) release microvesicles that transcriptionally reprogram injured cells, presenting protective effects against AKI, thereby modulating a specific miRNA–mRNA network (de Almeida et al., 2016), while the renoprotective effect of microvesicles was lost after treatment with RNase, non-specific miRNA depletion of microvesicles by Dicer knockdown in the progenitor cells, or transfection with specific miR- antagonists. Thus, the miRNAs in microvesicles contribute to reprogramming hypoxic resident renal cells to a regenerative program (Cantaluppi et al., 2012).

In the current study, we used next-generation sequencing to detect the content of miRNA in exosomes from USCs (USCs-Exos) and identified the abundant miR-216a-5p in exosomes. At the same time, we also studied the protective role of exosome miRNA transfer in I/R injury. It is suggested that miR-216a-5p can protect human proximal tubular epithelial cells (HK-2) against apoptosis by miRNA transfer, targeting the phosphatase and tensin homolog (PTEN) and activating Akt phosphorylation *in vitro*, while playing a role in recovering I/R damage *in vivo*.

## Materials and Methods

### Isolation, Proliferation, and Identification of USCs

Urine samples were collected from seven healthy adult male donors ranging in age from 22 to 28 years. USCs were collected, cultured, and identified as previously described (Guan et al., 2014). USCs collected from each individual were pooled together. After the urine samples were centrifuged, the resulting sediment was resuspended in Dulbecco’s modified Eagle medium (DMEM) supplemented with 2% (v/v) fetal bovine serum (FBS; Gibco, United States), 10 ng/ml of human epidermal growth factor (hEGF), 2 ng/ml of platelet-derived growth factor (PDGF), 1 ng/ml of transforming growth factor (TGF)-β, 2 ng/ml of basic fibroblast growth factor (bFGF), 0.5 μM of hydrocortisone, 25 μg/ml of insulin, 20 μg/ml of transferrin, 549 ng/ml of epinephrine, 50 ng/ml of triiodothyronine (T3), L-glu, and 20 IU/ml of antibiotics. Cell suspension was plated into cellular culture flasks and incubated at 37° in humidified atmosphere with 5% CO_2_.

The surface antigens of USCs were analyzed by a flow cytometer (FCM). USCs were stained with the positive markers CD29-PE, CD90-PE, and CD44-FITC and negative markers CD45-FITC, CD34-APC, and HLA-DR-PE. All antibodies were purchased from BD Biosciences (San Diego, CA, United States). USCs were incubated with antibodies for 30 min, then washed with PBS twice, and analyzed by flow cytometry using a FACSCanto II (BD Biosciences, San Jose, CA, United States). Isotype antibodies were used as controls.

### Isolation of USCs-Exos and MPs

Urine-derived stem cells were washed with PBS and cultured for an additional 24 h at 37°C and 5% CO_2_ in the exosome-free FBS. Exosomes and MPs are separated from USCs conditioned medium by continuous centrifugation, as described (Burger et al., 2015).

### Identification of USCs-Exos and MPs

*Transmission electron microscopy (TEM)* The exosome pellets were fixed in 3% (w/v) glutaraldehyde and 2% paraformaldehyde in cacodylate buffer, and then loaded to copper grids coated with Formvar. After washing, the grids were contrasted in 2% uranyl acetate, dried, and then examined by TEM (Morgagni 268D, Philips, Holland).

*Western blot* The specific surface markers: CD63, TSG101, and ALIX were used to confirm USCs-Exos.

*Nanoparticle tracking analysis* Size distribution and concentration of USCs-Exos and MPs were implemented by tunable resistive pulse sensing analysis which is a Nano platform with an NP100-rated nanopore (Ion Science, United Kingdom).

### Next-Generation Sequencing

The total RNA of USCs, USCs-Exos and MPs was used to prepare the template library. Two independent small RNA libraries were generated from USCs-Exos and MPs compared with the USCs library. Illumina Genome Analyzer IIx (Illumina Inc., San Diego, CA, United States) was used for sequencing.

### HK-2 H/R Model

HK-2 cells were cultured as a monolayer in DMEM/F12 medium, supplemented with 10% FBS, 1.0 mmol/L sodium pyruvate at 37°C in a humidified atmosphere containing 5% CO_2_. HK-2 cells were cultured in Hanks liquid without glucose and then treated in an anaerobic bag to mimic hypoxia model, HK-2 cells were treated with a high concentration of glucose (55 mmol/L) culture medium at 37°C in a humidified atmosphere containing 5% CO_2_ after ischemic treatment. HK-2 cells were treated with hypoxia for 1 h and reoxygenation for 24 h *in vitro* according to our previous studies (Cui et al., 2011; Zhang et al., 2016).

### Cell Counting Kit-8 (CCK8) Assay

A CCK8 assay (Beyotime Biotechnology Co., Ltd) was used for the cell proliferation assay. HK-2 cells were seeded in a 6-well plate and co-cultured with PBS, USCs-derived conditioned medium and exosomes. Ten microliters of CCK8 solution in fresh culture medium was added every 24 h and incubated for 2 h at 37°C, and the optical density (OD) value at 450 nm wavelength was determined using a microplate reader (Thermo Scientific, United States).

### miRNA Isolation and Real-Time PCR

Total RNA inclusive of the small RNA fraction was extracted using the miRNeasy Mini Kit (Qiagen Inc., Toronto, ON, Canada). Complementary DNA (cDNA) was synthesized using a Reverse Transcription System (Toyobo, Osaka, Japan) and real-time PCR was performed with SYBR Green PCR master mix (Applied Biosystems, Foster City, CA, United States) on an ABI 7500 fast real-time PCR system (Applied Biosystems, Carlsbad, CA, United States). Expression levels were normalized to the internal controls and the relative expression levels were evaluated using the 2^–Δ^ ^Δ^ ^*CT*^ method. The specific primers for miR-216a-5p were purchased from Genechem Ltd. (Genechem, Shanghai, China). The primer sequences are listed in Supplementary Table 1.

### Western Blot Analysis

Western blot analysis was performed according to routine protocols. Primary antibodies used were anti-CD63, anti-TSG101 (1:500; Santa Cruz Biotechnology, Santa Cruz, CA, United States); anti-PTEN, Akt, pAkt (1:1000; Cell Signaling Technology), and β-actin (1:1000; Abcam). Membranes were then incubated with secondary antibodies (1:10000) for 2 h at room temperature. The intensity of each band was analyzed using ImageJ software.

### Dual-Luciferase Reporter System

Dual-luciferase reported gene assay was employed to further assess whether PTEN was indeed a direct target gene of miR-216a-5p. Next, miR-216a-5p was co-transfected with reporter plasmids Pmir-PTEN-wild-type (WT) or pMIR-PTEN-mutant (MUT), respectively, into HEK-293T cells (CRL-1415, Shanghai Xinyu Biological Technology Co., Ltd). After 48 h, the cells were collected, lysed and centrifuged with the supernatant collected, then luciferase activity was assayed using a Dual Luminescence assay kit (RG005, Beyotime Biotechnology Co., Ltd). Reporter luciferase activity was normalized to the internal control Renilla luciferase activity in all samples.

### Caspase-3 Activity

Caspase-3 activity was measured using the Caspase 3 Activity Assay Kit (Beyotime Biotechnology Co., Ltd), as described (Sun and Yue, 2019).

### Rat Renal I/R Model and Injection of USCs-Exos

Rat renal I/R model was performed on adult male SD rats (Laboratory Animal Center of Peking University Health Science Center, China). We performed dorsal incisions to expose both the right and left kidneys. The left renal artery was separated and occluded with a non-traumatic microvascular clamp for 45 min to induce ischemia. The clamp was then removed and the kidney observed for 4–5 min to ensure that reperfusion was established successfully. The wounds were sutured and reperfusion continued for 24 h. Then the left kidneys were removed and sliced in half using a coronal cut, as described in our previous study (Zhang et al., 2015). 16 male SD rats were randomly divided into four groups: sham (*n* = 4), rats subjected to identical surgical procedures without renal arterial clamp; I/R (*n* = 4), rats underwent renal ischemia for 45 min followed by 24 h reperfusion and received the same volume of isotonic saline 15 min before the renal artery clamp was applied; I/R + USCs-Exos (*n* = 4), rats received an intravenous injection of USCs-Exos (20/40/80 μg) 15 min before the renal artery clamp was applied; I/R + USCs-Exos + miR-antagomirs (*n* = 4), rats received an intravenous injection of USCs-Exos transfected with the miR-216a-5p inhibitor miR-antagomirs 15 min before the renal artery clamp was applied.

### Histological Analysis

The renal Tissue specimens were embedded in paraffin and sliced into 4 μm-thick sections, stained with hematoxylin and eosin (H&E) according to standard procedures, and mounted on a glass slide. Two sections per kidney were evaluated under a standard light microscope. All samples were coded, and an experienced renal pathologist examined the specimens in a blinded fashion. Ten areas were randomly selected from the outer medulla kidney and observed at 200 magnifications. A renal pathologist performed a blind semiquantitative analysis of histologic lesions with scores ranging from 0 to 5, the maximum score per tubule was 5 and the higher scores indicated more severe damage. Pathological scoring ranged from 0 to 5 points based on the percentage of injury area as follows: 0, normal; 1, injury area < 10%; 2, injury area > 10% but < 25%; 3, injury area > 25% but < 50%; 4, injury area > 50% but < 75%; 5, injury area > 75%.

### Immunohistochemistry

Immunohistochemical staining for PTEN in the renal cortex was detected using commercial assay kits. We deparaffinized 4 μm-thick paraffin sections heated in citrate buffer, and then incubated them with primary antibodies against PTEN (rabbit anti-rat, 1:125, CST, United States). Subsequently, the sections were incubated with secondary antibodies for 30 min at 37°C. Color was developed using 3,30- diaminobenzidine tetrahydrochloride, followed by nuclear counterstaining with hematoxylin. Positive and negative controls were included in each step. Histomorphology was analyzed with an Eclipse E100 microscope (Nikon, Tokyo, Japan). The expression of PTEN was evaluated by calculating staining index. The staining index (values, 0–12) was determined by multiplying the score for staining intensity with the score for positive area. Ten visible areas from each sample were scored randomly in each section.

### TUNEL Assay

Apoptosis was assessed by the TUNEL assay using the *in situ* Cell Death Detection Fluorescein Kit (Beyotime Biotechnology Co., Ltd) according to the manufacturer’s protocol. Paraffin sections (4 μm) were deparaffinized, permeabilized with 20 mg/ml proteinase K at room temperature for 15 min and 0.2% Triton X-100/PBS for 15 min at 4°C, and then incubated with 3% H_2_O_2_, the sections were incubated with a mixture of nucleotides and TdT enzyme for 60 min at 37°C. The final count was expressed as the percentage of total cells detected by visualization under microscope. Finally, apoptotic cells were quantified as the average number of epithelial cells with bright green signals counted in 10 consecutive fields under 200 magnifications.

### Measurement of Renal Function

Serum creatinine and urea nitrogen levels were measured by AU5800 Analyzer (Beckman Coulter Ltd., CA, United States).

### Statistical Analysis

Data are reported as mean ± SEM and were analyzed using SPSS version 20.0 (IBM, Armonk, NY, United States). Comparisons between groups were performed using Mann-Whitney *U* test and One-way ANOVA. Graphic analyses were performed using GraphPad Prism 5.01 (GraphPad Software, San Diego, CA, United States). *P* value < 0.05 was considered to indicate a statistically significant difference.

### Ethics Statement

The present study was approved by the ethics committee of Peking University Third Hospital.

## Results

### Characterization of Human USCs

**FIGURE 1:**
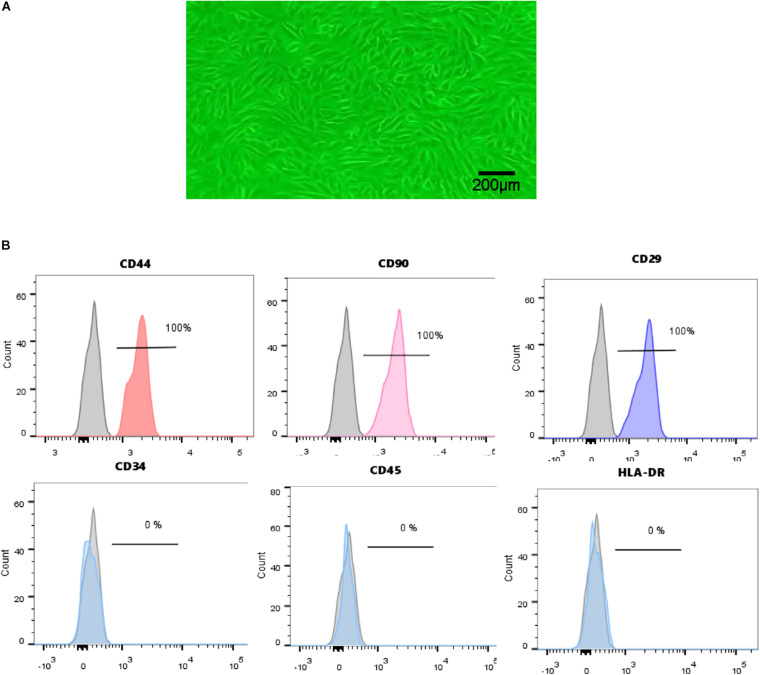
Characterization of human USCs. **(A)** The morphology of USCs was observed under a microscope. **(B)** Flow cytometric analysis of the expression of cell surface markers on USCs. USCs were positive for CD44, CD90, and CD29, and negative for CD34, CD45, and HLA-DR.

Urine-derived stem cells were isolated from freshly urine from 7 healthy male adults as described above. USCs in primary culture presented a fibroblast like morphology (Figure 1A). Cell surface markers were analyzed by FCM. USCs were positive for CD44, CD90, and CD29 and negative for CD34, CD45, and HLA-DR (Figure 1B). Based on the results above, we successfully isolated human USCs.

### Characterization of USCs-Exos and the Uptake of USCs-Exos by HK-2 Cells

**FIGURE 2:**
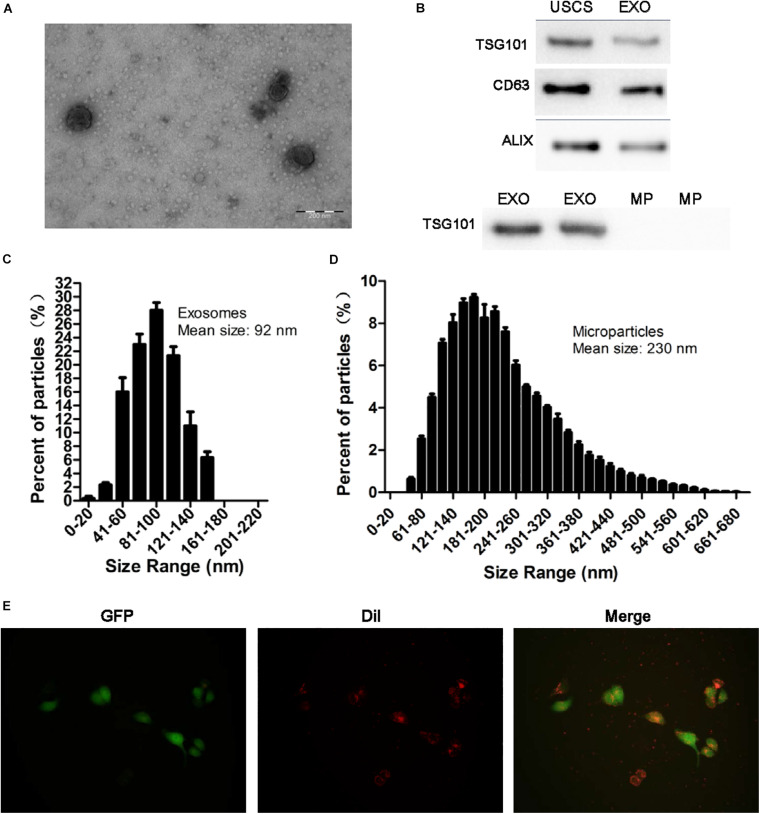
Identification of exosomes and the uptake of exosomes from USCs by HK-2 cells. **(A)** Morphology of USC-Exos under TEM (scale bar = 200 nm). **(B)** The expression of CD63, TSG101, and ALIX in exosomes, MPs and USCs was determined by Western blot analysis. **(C)** The mean diameter and the concentration of exosomes were measured by Nanoparticle tracking analysis. **(D)** The mean diameter and the concentration of MPs were measured by Nanoparticle tracking analysis. **(E)** Green fluorescent protein (GFP) was introduced into HK-2 cells, exosomes labeled with red fluorescent dye Dil are shown to be up take by HK-2 cells.

Transmission electron microscopy, NTA analysis and western blot were used to identify the nanoparticles derived from USCs. As shown in Figures 2A–D, exosomes were bilayer membrane vesicles, had a mean diameter of 92 nm and expressed TSG101, CD63, ALIX. Whereas the mean diameter of MPs was 230 nm, lacked of TSG101.

To further define whether USCs-Exos were taken up by HK-2 cells, a Dil dye was used to label the exosomes and then co-cultured with the target HK-2 cells. Fluorescence microscopy was used to monitor the situation of exosomes uptaken by HK-2 cells. As shown in Figure 2E, exosomes can be uptaken by HK-2 cells *in vitro*.

### USCs-Exos Play a Protective Role in HK-2 Cells Exposed to H/R

**FIGURE 3:**
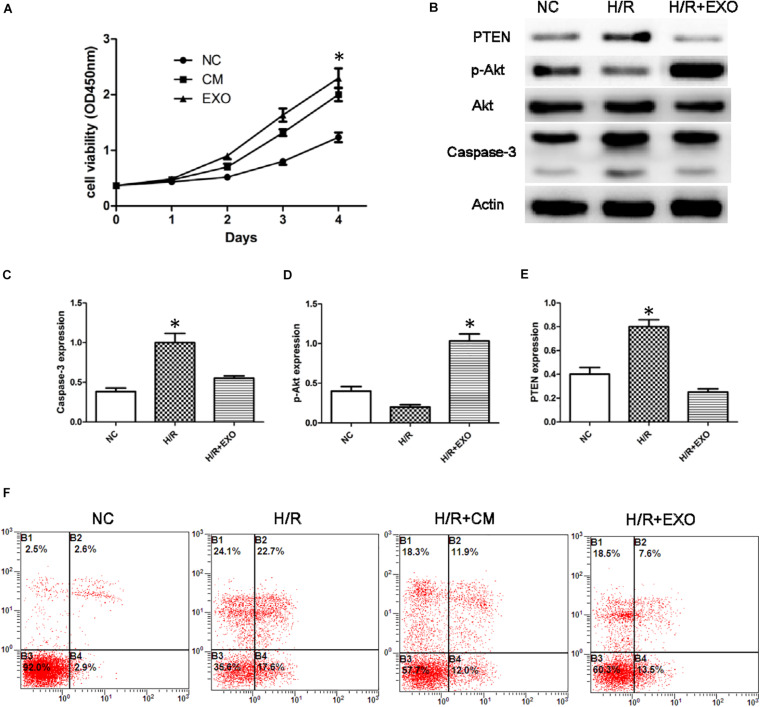
USCs-Exos play a protective role in the process of hypoxia/reoxygenation. **(A)** Cell proliferation of HK-2 cells measured by CCK8 assay after administration of USCs-derived conditioned medium(CM) and exosomes(EXO) (*n* = 3, two-way ANOVA). **(B)** Effect of USCs-Exos on the expression of PTEN and caspase-3 and phosphorylated Akt in HK-2 cells exposed to H/R. **(C–E)** Representative blots for caspase-3, pAkt/Akt, and PTEN are depicted. *P < 0.05 versus normoxic conditions, *n* = 3. **(F)** Representative FCM photograph of PI and annexin V–FITC double-stained podocyte apoptosis. *P < 0.05 versus H/R HK-2 cells and H/R + Exos or versus normoxic HK-2 cells and H/R.

To study the effect of USC-Exos in the H/R cell model, we employed CCK8 assay to detect the cell’s ability. The results showed that the proliferation of HK-2 cells subjected to H/R increased after administration with USCs-derived conditioned medium and exosomes (Figure 3A).

In HK-2 cells exposed to H/R, administration of USCs-Exos reduced the level of Caspase-3, stimulated Akt phosphorylation, and inhibited the expression of PTEN (Figures 3B–E). We then examined the role of USC-Exos in the protection against apoptosis by FCM. The number of early apoptotic cells increased after HK-2 suffered from H/R. The ratios of early apoptotic cells in the control and H/R groups were 2.6 and 22.7%, respectively. After the addition of USC-Exos (200 ng/ml), the ratio of early apoptotic cells in H/R group dropped to 7.6%. Similar results were obtained by USCs-derived conditioned medium (Figure 3F). These results indicated that USC-Exos played a protective role in the H/R process of HK-2 cells.

### Characterization of miRNAs in USCs-Derived Extracellular Vesicles

**FIGURE 4:**
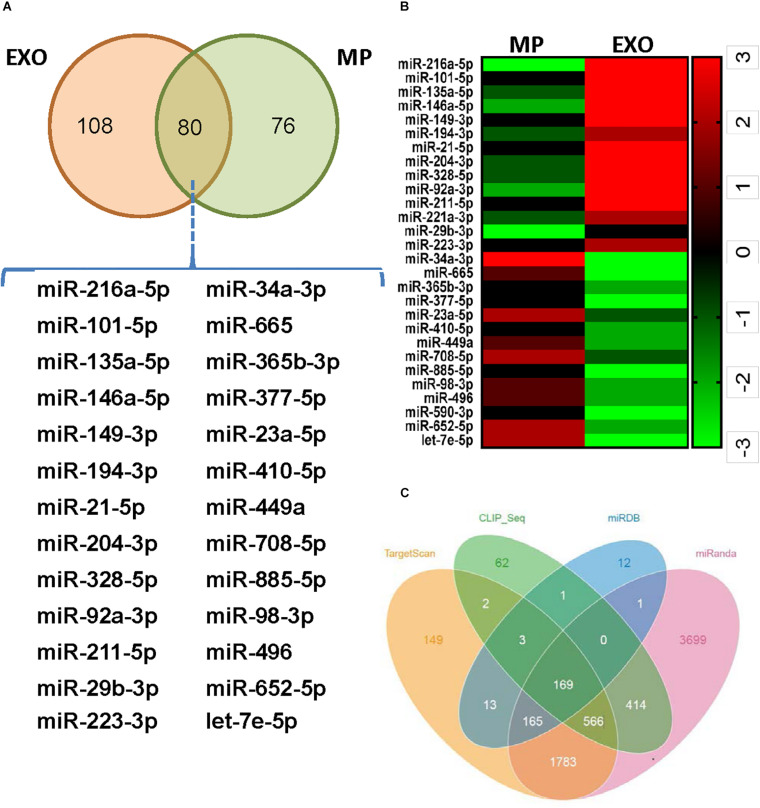
Exosomes from urine-derived stem cells (USCs-Exos) are highly enriched in miR-216a-5p. **(A)** Eighty miRNAs were co-expressed by exosomes and particles of urine-derived stem cells (USCs). **(B)** Heat map of the upregulated miRNAs and downregulated miRNAs with a ≥1.5-fold difference between USCs-Exos and microparticles (MPs). **(C)** Prediction of the target gene of miR-216a-5p; the comparisons of the predicted results are illustrated using a Venn diagram.

Exosomes mediate cell-to-cell communication by transferring their contents, especially miRNAs. USCs-Exos may play the regulating role by delivering exosomal miRNAs. miRNA libraries from USCs-Exos and MPs were characterized by Next Generation Sequencing, and the results showed that 80 miRNAs were co-expressed by USCs-Exox and MPs of USCs (Figure 4A). Cluster software was used to analyze 28 miRNAs with significant differences (Figure 4B), among which miR-216a-5p showed the most significant difference between the two groups. Furthermore, we used miRNA-gene relationship prediction websites, including miRDB, TargetScan, miRWalk CLIP-Seq and miRanda to predict the target gene of miR-216a-5p. The intersection of the target genes of miR-216a-5p was shown in various softwares using the Venn diagram (Figure 4C).

### MiR-216a-5p Protects HK-2 From H/R Injury and Targets PTEN

**FIGURE 5:**
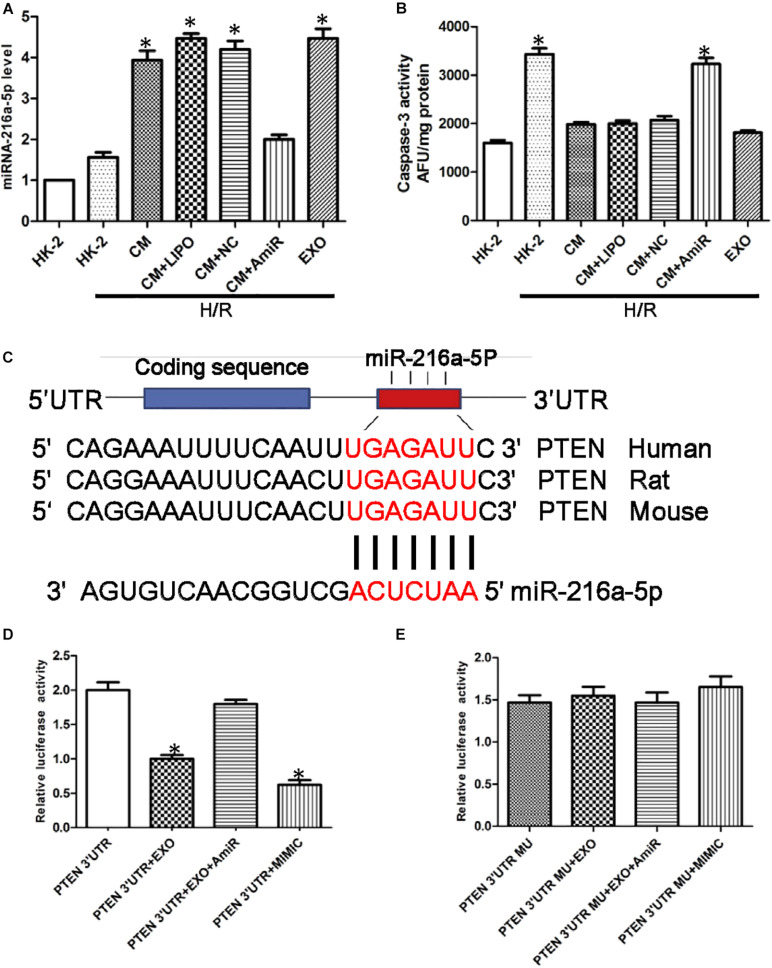
miR-216a-5p protects human proximal tubular epithelial cells (HK-2) from hypoxia/reoxygenation (H/R) injury and targets phosphatase and tensin homolog (PTEN). **(A)** Effect of USCs-derived conditioned medium (CM) or exosomes on miR-216a-5p levels in HK-2 cells exposed to H/R. LIPO, treatment with Lipofectamine (5 μl) alone, as a control group to exclude the effect of the transfection reagent on the experiment. NC, untreated HK-2 cells subjected to H/R. AmiR, treatment with the miR-216a-5p inhibitor (20 pmol/μl, 5 μl). *P < 0.05 versus HK-2 cells exposed to H/R, *n* = 3. **(B)** Caspase-3 activity in different groups. *P < 0.05 versus normoxic conditions (NC), CM/HR, CM + LIPO/HR, and CM + SCB/HR; *n* = 3. SCB (transfection with scrambled miRNA sequence, negative control). **(C)** The miRWalk software predicted PTEN might be the specific target gene of miR-216a-5p in HK-2 cells. **(D)** Effect of USCs-Exos (PTEN 3′UTR + EXO), co-transfection with the miR-216a-5p inhibitor (PTEN 3′UTR + EXO + AmiR), or a miR-216a-5p mimic (PTEN 3′UTR + MIMIC) on luciferase reporter activity. *P < 0.05 versus PTEN 3′UTR, *n* = 3. **(E)** Effect of transfection of a mutated construct of the 3′-UTR of PTEN (PTEN 3′UTR MU), *n* = 3.

The expression of miR-216a-5p in HK-2 cells was determined by RT-qPCR. In cultured HK-2 cells exposed to H/R, administration of USCs-derived conditioned medium (CM) or exosomes significantly increased the levels of miR-216a-5p, whereas conditioned medium from USCs transfected with the miR-216a-5p inhibitor blocked this increase (Figure 5A).

To verify the potential role of miR-216a-5p transferred from USCs-Exos in endothelial protection, HK-2 cells exposed to H/R were incubated in conditioned medium from USC, with or without miR-216a-5p antagomiR. As shown in Figure 5B, the activity of caspase-3 in the H/R treatment group was significantly increased, while USCs-derived conditioned medium and exosomes suppressed this effect. On the other hand, conditioned media from USCs transfected with miR-216a-5p antagomiR did not affect the increase in caspase-3 activity caused by H/R exposure.

To explore the direct target of miR-216a-5p in HK-2 cells, we screened the target gene of miR-216a-5p using the miRWalk software and found that miR-216a-5p could directly bind to PTEN’s 3′UTR region, implying PTEN might be the specific target gene of miR-216a-5p in HK-2 cells (Figure 5C). Combined with dual luciferase reporter assay, we discovered that the luciferase reporter activity of PTEN 3′UTR was obviously inhibited in the groups of USCs-Exos and miR-216a-5p mimic treated cells. Whereas the inhibitory effect of USCs-Exos on luciferase activity was blocked when HK-2 cells were transfected with miR-216a-5p antagomiR (Figure 5D). On the contrary, neither USCs-Exos nor transfection of HK-2 cells with a miR-216a-5p mimic reduced luciferase activity when HK-2 cells were transfected with a mutated vector of the PTEN 3′UTR (Figure 5E).

### USCs-Exos Play a Protective Role by Regulating the miR-216a-5p/PTEN/Akt Signaling Pathway

**FIGURE 6:**
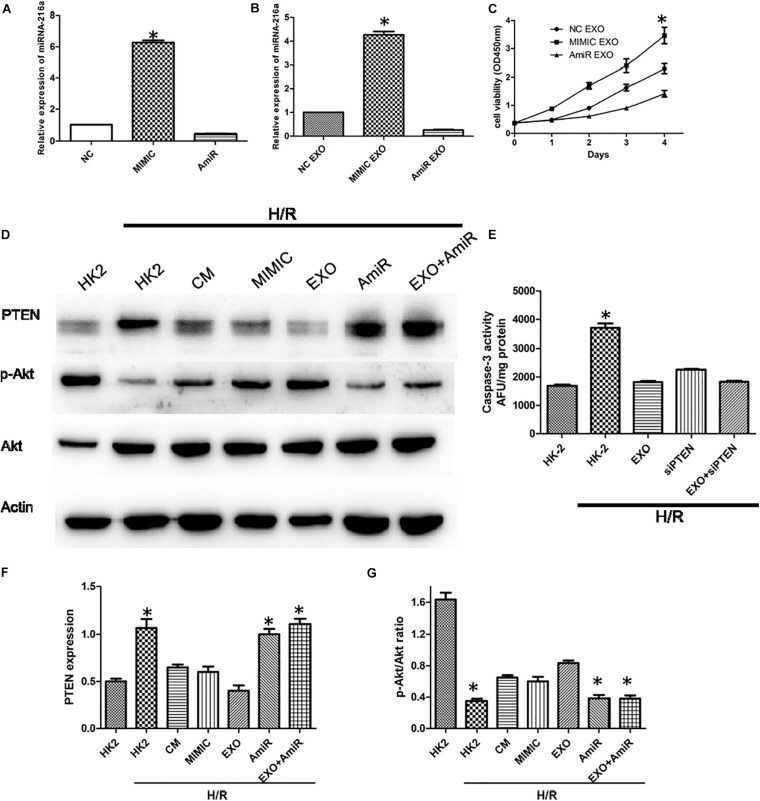
USCs-Exos play a protecting role by regulating the miR-216a-5p/PTEN/AKT signaling pathway. **(A)** The expression of miR-216a-5p in HK-2 cells treated with the miR-216a-5p mimic and inhibitor by real time PCR. *P < 0.05 versus PBS control, *n* = 3. **(B)** The expression of miR-216a-5p in HK-2 cells treated with exosomes derived from USCs transfected with the miR-216a-5p mimic and inhibitor. *P < 0.05 versus PBS control, *n* = 3. **(C)** Cell proliferation of HK-2 cells measured by CCK8 assay after administration of exosomes derived from USCs transfected with the miR-216a-5p mimic and inhibitor. *P < 0.05 versus PBS control, *n* = 3. **(D)** Expression of PTEN and phosphorylation of Akt by Western blot. **(E)** Graph depicts caspase-3 activity in HK-2 cells exposed to H/R, with or without transfection with siRNA to PTEN, and with or without USCs-Exos.*P < 0.05 versus all other groups; *n* = 3. **(F)** Representative blots for PTEN and Actin are depicted. Five microliters of mimic and anti-miR with concentration of 20 pmol/μl were added, respectively. *P < 0.05 versus normoxic conditions, *n* = 3. **(G)** Representative blots for phosphorylated Akt and total Akt are depicted. *P < 0.05 versus normoxic conditions, *n* = 3.

In order to determine whether miR-216a-5p could be transferred into HK-2 cells from USCs through exosomes, the expression of miR-216a-5p in HK-2 cells treated with the miR-216a-5p mimic and inhibitor were determined by RT-qPCR. In the meantime, the miR-216a-5p levels in HK-2 cells administrated with USCs-Exos transfected with the miR-216a-5p mimic and inhibitor were measured. The results showed that the expression of miR-216a-5p in HK-2 cells was significantly increased after treatment with miR-216a-5p mimics (Figure 6A) and USCs-Exos transfected with miR-216a-5p mimic (Figure 6B) compared with the levels in the groups administrate with the miR-216a-5p inhibitors. The results above indicated that the levels of miR-216a-5p could be transferred to HK-2 cells though USCs-Exos. CCK8 assays were used to assess the proliferation of HK-2 cells treated with USCs-Exos transfected with the miR-216a-5p mimic and inhibitor, respectively. The results showed that the proliferation of HK-2 cells subjected to H/R treated with USCs-Exos of overexpressed miR-216a-5p was significantly higher than those treated with negative control and miR-216a-5p knockdown USCs-Exos (Figure 6C), suggested that the overexpression of miR-216a-5p in USCs-Exos can promote the proliferation of renal tubular epithelial cells *in vitro*.

To further illustrate the role of miR-216a-5p/PTEN signaling on USCs-Exos-mediated damage repair, we treated HK-2 cells exposed to H/R with USCs-derived conditioned medium, exosomes, miR-216a-5p mimic and inhibitor. As shown in Figures 6D,F,G, H/R stimulated PTEN expression was blocked by conditioned medium and USCs-Exos and miR-216a-5p mimic, while Akt phosphorylation in HK-2 cells subjected to HR were activated. By contrast, USCs-Exos transfected with the miR-216a-5p inhibitor failed to inhibit HR-mediated increases in PTEN and declines in Akt phosphorylation.

To clarify the role of PTEN in apoptosis, HK-2 cells were treated with siRNA to reduce PTEN expression before exposure to H/R and treatment with USCs-Exos. As shown in Figure 6E, silencing of PTEN eliminated the activation of caspase-3 in HK-2 exposed to HR. In addition, in HK-2 cells knockdown of PTEN, exosomes further inhibited caspase-3 activation.

In a word, miR-216a-5p/PTEN/Akt might be the main signaling pathway in the process of USCs-Exos -mediated damage repair in HK-2 cells subjected to H/R.

### *In vivo* Effect of USCs-Exos and miR-216a-5p on Kidney I/R Injury in Rats

**FIGURE 7:**
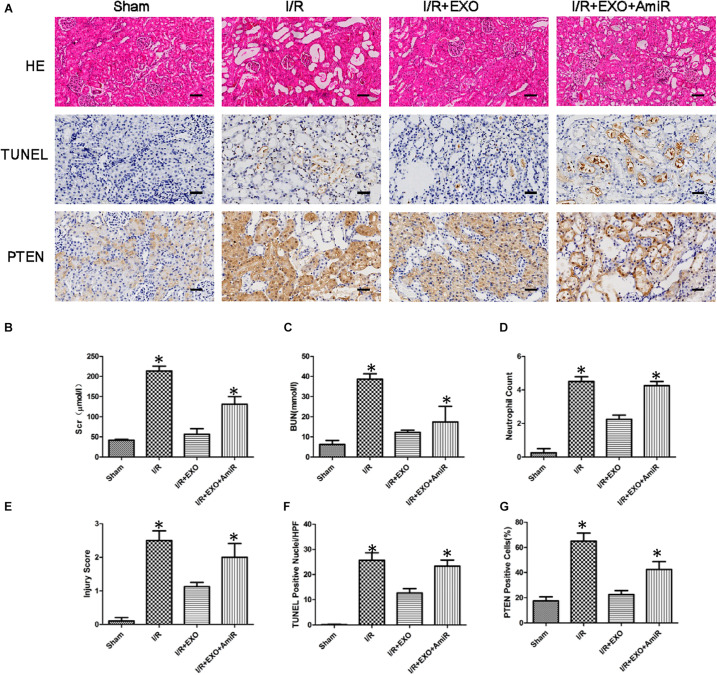
Effect of exosomes derived from urine-derived stem cells (USCs) with or without previous transfection with the miR-216a-5p inhibitor on renal function, histologic injury, apoptosis, and phosphatase and tensin homolog (PTEN) in rats with ischemia/reperfusion (I/R) injury. **(A)** Representative photomicrographs of renal histology with hematoxylin-and-eosin (H&E) staining, transferase-mediated deoxyuridine triphosphate-biotin nick end labeling (TUNEL) staining, and PTEN expression by immunohistochemical staining in sham-treated SD rats, SD rats with kidney I/R injury, SD rats with kidney I/R injury treated with USC-Exos, and SD rats with kidney I/R injury treated with exosomes derived from USCs transfected with the miR-216a-5p inhibitor. Original magnification × 200. Scale bar = 30 μm. **(B)** Graph shows plasma creatinine levels in the four groups of SD rats 24 h after I/R. *P < 0.05 versus sham, *n* = 4. **(C)** Graph shows plasma urea levels in the four groups of SD rats 24 h after I/R. *P < 0.05 versus sham, *n* = 4. **(D)** Graph shows kidney neutrophil infiltration in the four groups of SD rats depicted as numbers of neutrophils per high-power field. *P < 0.05 versus sham, *n* = 4. **(E)** Graph shows histologic kidney injury scores in the four groups of SD rats. *P < 0.05 versus sham, *n* = 4. **(F)** Graph depicts semiquantitative assessment of TUNEL staining in kidneys of the four groups of SD rats depicted as TUNEL-positive nuclei per high-power field. *P < 0.05 versus sham, *n* = 4. **(G)** Graph shows the quantification of the percentage of PTEN expression of the four groups of SD rats.

We used a renal I/R injury rat model to validate whether USCs-Exos and miR-216a-5p can improve the impaired renal function *in vivo*. SD rats received an intravenous injection of PBS, USCs-Exos, and USCs-Exos transfected with the miR-216a-5p inhibitor 15 min before the renal artery clamp was applied. The serum creatinine (Cr) and urea levels in the I/R group were significantly higher than those of the sham group (Figures 7B,C). However, the Cr and urea levels were significantly reduced in rats pretreated with USCs-Exos. Consistent with the kidney functional tests, we found histopathological features such as karyolysis, karyorrhexis, vacuolar degeneration, atrophy, tubular structure degeneration, tubular necrosis, and neutrophil infiltration in rats with kidney I/R injury. As shown in Figures 7A,D,E,F, administration of USCs-Exos to SD rats with I/R potently protects against kidney injury, as evidenced by alleviated histologic injury, decreased infiltration of neutrophils, and significantly reduced number of TUNEL-positive cells. At the same time, we tried to use 20, 40, and 80 μg of exosomes to deal with the model, but there is no dose–effect relationship (Supplementary Figure 1). Our results demonstrated that 20 μg exosomes have already shown a protective effect. However, the protective effect did not further increase if we increase the dose of exosomes to 40 and 80 μg. Furthermore, USCs-Exos inhibited kidney PTEN expression compared with I/R alone. The protective effects were weakened in the rats receiving USCs-Exos transfected with the miR-216a-5p inhibitor (Figures 7A,G).

## Discussion

Ischemic injury is the main cause of AKI, and there is a significant lack of therapeutic options for treatment. Previous research has shown that administration of USCs was able to improve renal function and histological damage, promote proliferation, reduce apoptosis of renal tubular epithelial cells, and inhibit inflammation in the AKI model (Tian et al., 2017; Sun et al., 2019). USCs as novel stem cells can be conveniently obtained from urine through non-invasive methods, which have the properties of extensive self-renewal and pluripotent differentiation. They are homologous with the urinary system and do not induce immune rejection. Therefore, USCs are considered an ideal cell source for the treatment of AKI. Studies have shown that the therapeutic effect of transplantation of USCs may be related to its paracrine mechanism. USCs-Exos have been shown to be therapeutic for a variety of diseases as compared to direct use of USCs (Jiang et al., 2016; Ling et al., 2019). As a potential therapeutic option, exosomes may also overcome the limitations of direct USCs transplantation. In this study, we investigate the protective role of the USCs-Exos *in vitro* and *in vivo*, the results showed that USCs-Exos increase the proliferation and inhibit the apoptosis of HK-2 cells, indicate that USCs-Exos may play a protective role in the process of renal I/R injury of AKI. However, the relevant mechanism for its role is still unclear.

Some studies have reported that exosomes derived from stem cells play their biological functions on target cells by the delivery of specific miRNAs. Microvesicles derived from endothelial progenitor cells protect the kidney from ischemic acute injury by delivering miRNAs, such as miR-126 and miR-296 (Cantaluppi et al., 2012). Jose L. et al. revealed exosomes from human cord blood endothelial colony forming cells transferred miR-486-5p to endothelial cells, targeting of PTEN/Akt pathway, protected against ischemic injury in AKI (Vinas et al., 2016). Wang C. et al. (2019) evidenced that miR-199a-5p, which was abundant in the BMSCs, was transferred into renal tubular epithelial cells in a time-dependent manner and significantly inhibited I/R-induced ER stress by targeting binding immunoglobulin protein.

In the present study, we confirmed that miR-216a-5p in USCs-Exos can be taken up by renal tubular epithelial cells and reduce their apoptosis through regulating the PTEN/Akt pathway. USCs-Exos may be a new therapeutic tool for AKI treatment. This conclusion is supported by the following results: (1) USCs-Exos were rich in miR-216a-5p, and delivery of exosomes to cultured HK-2 cells exposed to H/R injury could increase miR-216a-5p level, inhibit PTEN expression and enhance Akt phosphorylation. (2) USCs-derived conditioned medium and exosomes can inhibit H/R-induced apoptosis, which is related to PTEN expression inhibition and Akt activation, and this effect was blocked by the miR-216a-5p inhibitor. (3) miR-216a-5p directly targets PTEN in HK-2 cells, and silence of PTEN prevented H/R-induced cell apoptosis. (4) USCs-Exos effectively protect rats from renal I/R injury. This effect disappeared when USCs first transfected with antagomiR to miR-216a-5p.

Renal tubular epithelial damage is the main pathological event of AKI, and the therapeutic strategy to minimize the apoptosis of renal tubular epithelial cells in AKI has become the subject of extensive research (Linkermann et al., 2014). Therefore, we selected HK-2 as major targets of exosomal transfer of miR-216a-5p after ischemic injury. We found that miR-216a-5p from USCs-Exos inhibited renal tubular epithelial cell apoptosis by targeting PTEN and regulating the Akt pathway. However, endothelial cell dysfunction may also be very important in ischemic AKI. The reduction of renal blood flow will not only reduce the oxygen supply to renal tubular cells, causing further damage, but also prevent the delivery of drugs to renal tubular cells, thereby weakening the therapeutic effect. This study does not clarify the final location of USCs-Exos in the kidney. Whether USCs can play a role in damaged renal tubular epithelial cells and endothelial cells is worthy of further study.

Some stem cell-derived extracellular vesicles microRNAs are reported to be important for AKI therapy (Wang S.Y. et al., 2019) including miRNA-126 and miRNA-296 in EVs from endothelial progenitor cells (Cantaluppi et al., 2012), miR-486-5p from ECFC exosomes (Vinas et al., 2016) and miR-218 from termed renal artery-derived vascular progenitor cells exosomes (Pang et al., 2017). In the current study, It has been determined that miR-216a-5p was rich in exosomes and plays a central role in AKI treatment. MiR-216a-5p has been reported to be involved in the progression of suppressing inflammation and promoting cell proliferation (Huang et al., 2019; Peng et al., 2019). A research has reported a negative correlation between the levels of miR-216a-5p and inflammatory cytokines (Peng et al., 2019). We co-cultured the USCs-Exos with HK-2 cells under the process of H/R. The results above indicated that the miR-216a-5p could be transferred to HK-2 cells though USCs-Exos. Studies on the proliferation of HK-2 cells with overexpression and knockdown of miR-216a-5p showed miR-216a-5p in USCs-Exos played the protective role under the process of H/R. We will further investigate the role of the immune system in this I/R process in our later studies.

We further explored the pathway miR-216a-5p regulated, we utilized miRWalk software which predict PTEN might be the specific target gene of miR-216a-5p in HK-2 cells. The role of PTEN in promoting apoptosis and inhibiting proliferation has been demonstrated in some researches. The PI3K/PTEN/AKT signaling pathway is essential to protecting renal tubular epithelial cells from apoptosis induced by cisplatin and H/R (Chen et al., 2019; Zhang et al., 2019). In this study, a luciferase reporter gene assay confirmed that PTEN is a direct target of miR-216a-5p and is negatively regulated by miR-216a-5p. Silencing PTEN with small interfering RNA can significantly reduce the effect of miR-216a-5p inhibition on hypoxia-induced apoptosis of HK-2 cells. We also observed that in HK-2 cells PTEN knockdown, USCs-Exos treatment could further inhibit the activity of caspase-3. We speculate that in addition to PTEN, other potential targets of miR-216a-5p should be considered in the overall protection response. Alternative targets for miR-216a-5p include the transcription factor FoxO1, which is related to the human mesangial cell proliferation in diabetic nephropathy, and BAX, which is associated with the protection neurons from apoptosis in Parkinson’s disease (Yang et al., 2020).

In the current study, 20 μg of USCs-Exos was delivered to rats 15 min before kidney I/R injury. We found that the expression of PTEN and apoptosis were decreased, and the renal function was improved.

However, there are still some limitations in our present study. Although the location and content of miR-216a-5p in HK-2 cells and kidneys of the rats have been detected by fluorescence *in situ* hybridization, we did not determine the final location of USC-Exos in rats due to the limitations of our experimental conditions, this part of experiment might be addressed in future studies.

**FIGURE 8:**
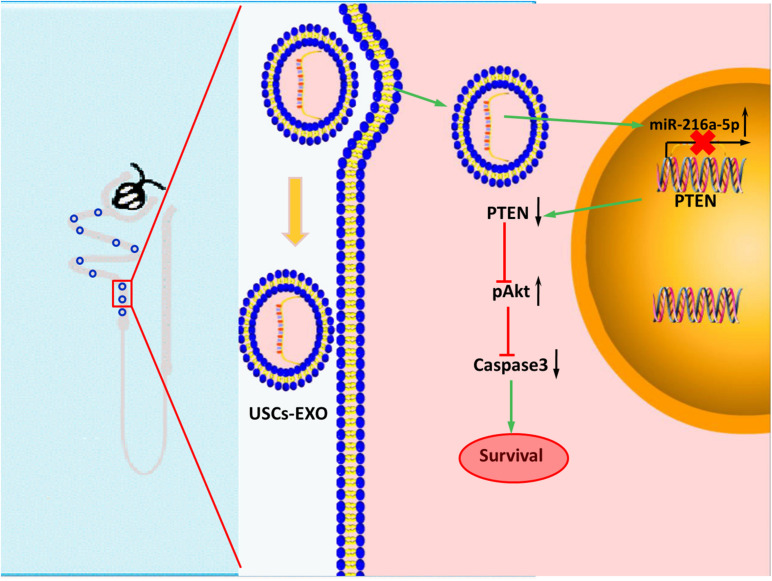
Transfer of microRNA-216a-5p from exosomes secreted by human urine-derived stem cells reduces renal ischemia/reperfusion injury.

In conclusion, miR-216a-5p carried in USCs-Exos could transfer to renal tubular epithelial cells and relieve cell damage in HK-2 cell exposed to H/R and renal I/R injury rat model by targeting PTEN/Akt pathway (Figure 8). Potentially, USCs-Exos can serve as a promising therapeutic option for AKI.

## Data Availability Statement

The original contributions presented in the study are included in the article/Supplementary Material, further inquiries can be directed to the corresponding authors.

## Ethics Statement

The studies involving human participants were reviewed and approved by the Peking University Third Hospital Medical Science Research Ethics Committee. The patients/participants provided their written informed consent to participate in this study. The animal study was reviewed and approved by the Peking University Third Hospital Medical Science Research Ethics Committee.

## Author Contributions

JW, SY, and LC conceived and contributed to design of the study. SY, YZ, JW, BY, RQ, AL, and HG performed the experiments. JD, HL, HY, and DW analyzed and interpreted the data. YZ, JW, and SY supervised and contributed to writing the manuscript. All authors read and approved the final manuscript.

## Conflict of Interest

The authors declare that the research was conducted in the absence of any commercial or financial relationships that could be construed as a potential conflict of interest.

## Abbreviations

AKI: acute kidney injury
CCK8: Cell Counting Kit-8
CM: conditioned medium
ECFC: endothelial colony-forming cell
FBS: fetal bovine serum
FCM: flow cytometer
H&E: hematoxylin and eosin
HK-2: human proximal tubular epithelial cells
H/R: hypoxia/reoxygenation
I/R: ischemia/reperfusion
miRNA: microRNA
MPs: microparticles
MSCs: mesenchymal stem cells
MUT: mutant
PTEN: phosphatase and tensin homolog
RNase: ribonuclease
TEM: transmission electron microscopy
TUNEL: transferase-mediated deoxyuridine triphosphate-biotin nick end labeling
USCs: urine-derived stem cells
USCs-Exos: exosomes from urine-derived stem cells
UTR: untranslated region
WT: wild type.
