# Supplementary material for: Transfer of MicroRNA-216a-5p From Exosomes Secreted by Human Urine-Derived Stem Cells Reduces Renal Ischemia/Reperfusion Injury
Source: Front Cell Dev Biol. 2020 Dec 22;8:610587. doi: 10.3389/fcell.2020.610587 (PMC7783217; doi:10.3389/fcell.2020.610587)
Supplement: Supplementary file 1 [file Data_Sheet_1.docx]

**Supplementary Table 1. Sequences of the primers**

| Primer | Sequence (5'-3') |
| --- | --- |
| Has-miR-216a-5p | F, CTCAACTGGTGTCGTGGAGTCGGCA  ATTCAGTTGAGT |
|  | R, ACACTCCAGCTGGGTAATCTCAGCTGGCAA |
| U6 | F,CTCGCTTCGGCAGCACATATACT |
|  | R, ACGCTTCACGAATTTGCGTGTC |
| Has-miR-216a-5p mimics | F, UAAUCUCAGCUGGCAACUGUGA  R, ACAGUUGCCAGCUGAGAUUAUU |
| Negative control | F, UUCUCCGAACGUGUCACGUTT  R, ACGUGACACGUUCGGAGAATT |


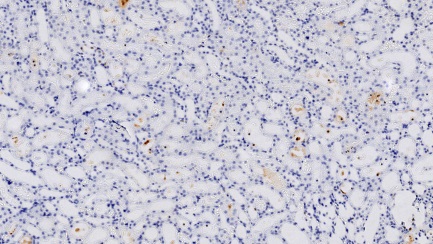

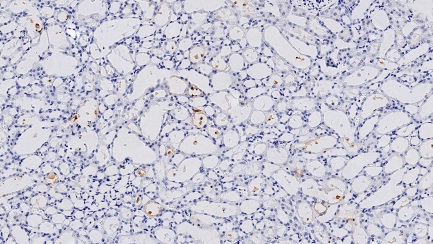

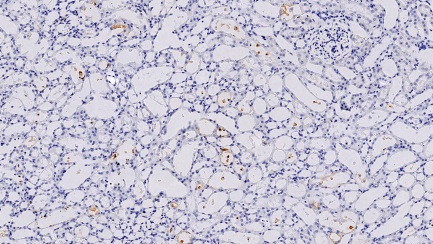


**TUNEL**

**I/R +EXO (20ug) I/R+EXO(40ug) I/R+EXO(80ug)**

**supplement Figure1 Effect of USCs exosomes dose on the protection of IRI kidney model**

Representative photomicrographs of renal histology with TUNEL staining by immunohistochemical staining in SD rats with kidney I/R injury treated with different dose of USC-Exos. Original magnification ×200. Scale bar=30 μm.
